# Preparation and *In Vitro* Evaluation of Ethylcellulose and Polymethacrylate Resins Loaded Microparticles Containing Hydrophilic Drug

**DOI:** 10.1155/2014/904036

**Published:** 2014-04-10

**Authors:** Satish Pandav, Jitendra Naik

**Affiliations:** Department of Pharmaceutical Technology, University Institute of Chemical Technology, North Maharashtra University, Jalgaon 425 001, India

## Abstract

*Objective*. The purpose of the recent study was to prepare and estimate sustained release of Ethylcellulose (300 cps) and Eudragit (RS 100 and RL 100) microparticles containing Propranolol hydrochloride used as a treatment of cardiovascular system, especially hypertension. *Method.* Propranolol hydrochloride was microencapsulated with different polymers (Ethylcellulose, Eudragit RS, and Eudragit RL) using modified hydrophobic (O/O) solvent evaporation method using 1 : 1 combination of acetone and isopropanol as the internal phase. Obtained microparticles were showing higher batch yield with higher encapsulation efficiency. Microparticles were prepared with different ratios of 1 : 1, 1 : 3, 1 : 5, and 1 : 7 (%, wt/wt) using span 80 (%, v/v) as a surfactant. *Results*. The influence of formulation factors like drug: polymer ratio, internal phase, and type of polymers on obtained microparticles was characterized with respect to particle size distribution, encapsulation efficiency, percentage yield, FTIR, and FE-SEM. Higher encapsulation efficiencies were obtained with various polymers like Ethylcellulose (96.63 ± 0.5) compared to Eudragit RS 100 (83.70 ± 0.6) and RL 100 (89.62 ± 0.6). The *in vitro* release study was characterized by initial burst. *Conclusion*. The result of study displays that Ethylcellulose and Eudragit loaded microparticles of Propranolol hydrochloride can be effectively prepared using modified hydrophobic emulsification solvent evaporation technique. Therefore, the modified hydrophobic emulsion technique can also be applied to the preparation of microparticles for low molecular weight and highly water soluble drugs.

## 1. Introduction

Ethylcellulose, a nonbiodegradable and biocompatible polymer, one of the extensively studied encapsulating materials for the controlled release of pharmaceuticals, was selected as the retardant material for Propranolol hydrochloride. Ethylcellulose, a polymer to microencapsulate a drug by coacervation phase separation technique, emulsion solvent evaporation technique, and spherical crystallization technique [1]. Eudragit RS and Eudragit RL are biocompatible copolymers synthesized from acrylic and methacrylic acid ester having similar structure that differs only in the extent of the quaternary ammonium substitution and hence high water permeability and hydrophilicity in Eudragit RL as compared to Eudragit RS polymer [2]. Propranolol hydrochloride exhibits short biological half-life (2-3). It is a beta blocker and commonly used for the treatment of hypertension which affects nearly about 10–20% of the population [3, 4]. The purpose of the present work was to prepare and evaluate oral sustained release microparticulate drug delivery system of Propranolol hydrochloride using three different forms of polymers such as Ethylcellulose, Eudragit RS, and Eudragit RL and to evaluate the drug release from microparticles prepared by hydrophobic emulsion solvent evaporation method (O/O) with a high entrapment capacity and extended release of the drug [5, 6]. Various process and formulation parameters such as a drug polymer ratio and surfactant concentration were optimized to maximize the entrapment efficiency of the drug. These microparticles were evaluated for encapsulation efficiency, drug content, and* in vitro* drug release. Drug-polymer interactions in the solid state were studied by Fourier transform infrared spectroscopy (FTIR); the size and shape were evaluated by Motic microscope. The aim of the current work was to encapsulate Propranolol hydrochloride with Ethylcellulose, Eudragit RS 100, and RL 100 microparticles that were prepared by modified hydrophobic solvent evaporation techniques. 1 : 1 combination of acetone and isopropanol was used as an internal phase and paraffin oil as external phase [7]. Prepared microparticles were characterized for drug content, particle size,* in vitro* drug release, and kinetic release studying.

## 2. Materials and Methods

### 2.1. Materials

The highly water soluble drug, Propranolol HCl, was gift samples from Micro Advanced Research Center, Pvt. Ltd., Bangalore, and used as a model drug. The nonbiodegradable polymer, Ethylcellulose (300 cps) was purchased from Sigma Eldritch, USA. The two biodegradable polymers, Eudragit RS and Eudragit RL, were donated by Colorcon Asia Pvt. Ltd., Goa. Acetone, isopropane, and N-Hexane were from Merck Specialities Pvt. Ltd., Mumbai. Heavy liquid paraffin, potassium dihydrogen phosphate, and sodium hydroxide were procured from S.D. Fine Chemicals, Mumbai, India.

### 2.2. Preparation of Microparticles

Polymeric microparticles were prepared by dispersing accurately weight quantities of Propranolol hydrochloride and polymers individually (Ethylcellulose 300 cps, Eudragit RS 100, and RL 100) in the primary phase as a solvent (1 : 1 combination of acetone and isopropanol) with continuous stirring at 500 rpm by using magnetic stirrer for 15 min. Sustained released microparticles of drug were prepared by modified hydrophobic emulsion solvent evaporation method (O/O) [8]. This primary emulsion was slowly added to the external secondary oil phase containing span 80 (0.4% v/v) as an emulsifying agent with constant stirring for 2 hours using a four-blade lab stirrer (Remi Elektrotechnik Ltd., Mumbai) at a speed of 1000 rpm. After complete evaporation, stirring was stopped, the n-hexane (20 mL) was added to harden the formed microparticles, and the mixture was vacuum filtered to obtain microparticles. The resulting microparticles were collected and allowed to dry for 24 h at room temperature. All batches of prepared microparticles are in triplicate way and all results are articulated as the mean value of three inspections.

### 2.3. Determination of Mean Particle Size

The diameter of microparticle of each formulation was measured by spreading a thin layer of microparticles on a glass slide and viewing the microparticles under an optical microscope fitted with an eyepiece having 40x to 100x resolution of Motic microscope (B1 series, Motic, China). Each sample was measured at three times and an average particle size was articulated as mean diameter.

### 2.4. Determination of Encapsulation Efficiency

The encapsulation efficiency of the prepared microparticles was determined by accurate weighing and added in acetone to dissolve polymer and then add the required volume of distilled water. Precipitate solution was filtered and make up the volume up to 100 mL into a volumetric flask. Recovered filteration was measured at maximum absorbance at 289 nm by using UV-visible spectrophotometer (UV-1800 Shimadzu Co. Ltd., Japan) and encapsulation efficiency was calculated using the following equation:

(1) $$\begin{array}{c} \text{EE}\;\left(\%\right)=\frac{\left(\text{Actual}\;\text{amount}\;\text{of}\;\text{drug}\right)}{\left(\text{Theoretical}\;\text{amount}\;\text{of}\;\text{drug}\right)}\times 100. \end{array}$$

### 2.5. Drug Polymer Interaction by FTIR

IR spectra of pure drug and microparticles prepared by using polymers like Ethylcellulose, Eudragit RS, and Eudragit RL-loaded microparticles were obtained with infrared (IR) spectra of the samples that were scanned in the range from 400 to 4000 cm^−1^ and recorded on a Fourier transform infrared spectrometer (Shimadzu Co. Ltd., Singapore), using the KBr disk technique.

### 2.6. Scanning Electron Microscopy

The surface morphology of microparticles was analyzed by scanning electron microscopy (SEM). The microparticles were fixed with carbon-Glu and coated uniformly with gold palladium under argon atmosphere. Samples were then observed with a Hitachi model S4800, Japan, scanning electron microscope.

### 2.7. *In Vitro* Drug Release Study

*In vitro* dissolution studies were performed using USP Type II dissolution test apparatus (Paddle) at 100 revs./min and temperature at 37°C ±  0.5°C. Withdrawing 5 mL samples at preselected time intervals up to 12 hours monitored progress of the dissolution. The same volume of dissolution medium was replenished after each sampling. The absorbance of the withdrawn samples was assayed by UV-spectophotometry at the wavelength of maximum absorbance (289 nm). Each dissolution study was carried out in triplicate and the mean values were expressed as ± standard deviation.

### 2.8. Kinetics of Drug Release

The* in vitro* drug release data was analysed according to zero order, first order, Higuchi square root, Hixon crowel, and Korsemeyer model. Selecting the most suitable model was preferred on the basis of integrity of fit test [9–12].

## 3. Results and Discussion

In the present paper, Ethylcellulose (300 cps) polymer has higher viscosity grade compared with polymethacrylate resins polymers like Eudragit RS 100 (RS) and RL 100 (RL). These are two copolymers synthesized from acrylic and methacrylic acid esters, containing a low level of quaternary ammonium groups. RS has a lower content of charged groups (4.5–6.8%), and it is considered less permeable to water with respect to the more readily permeable RL (8.8–12% ammonium groups) [13]. Higher permeability of Eudragit RL 100 is due to maximum number of quaternary ammonium substitution present in the structure of Eudragit RL 100 compared to RS 100 which affects the release behavior of the drug [14]. In this work, the effect of drug-polymer ratio and surfactant concentrations on particle size, entrapment efficiency, and release pattern of Propranolol hydrochloride from Ethylcellulose, Eudragit RS 100, and RL 100 microparticles prepared by nonaqueous (hydrophobic) solvent evaporation method was examined. Due to decrease in solubility of propranolol hydrochloride in external oil phase, the drug loss was reduced and enhanced the encapsulation efficiency. In this study we used Ethylcellulose polymer having 300 cps viscosity range as drug carrying polymer. Due to the high viscosity range it formed a saturated solution with acetone: isopropane organic solvent [15]. Ethylcellulose was hydrophobic in nature; thus, the hydrophobic polymer encapsulates larger amount of the drug. When organic phase was added in external oil phase containing surfactant, PROPA-EC matrix immediately starts to precipitate because of insoluble in oil and fast diffusion of acetone. One of the objectives of nonaqueous emulsion technique was to entrap maximum amount of Propranolol hydrochloride [16]. Due to polymer saturated solvent and 1 : 1 combination of acetone and isopropane immiscible with oil, polymeric matrix was immediately precipitated out as solvent starts to evaporate and gives maximum encapsulation efficiency. Span 80 is a better surfactant in terms of encapsulation efficiency, drug content, and particle size. Span 80 has greater propensity to migrate toward the surface of Ethylcellulose microparticles. So saturated solution of polymer (EC 300 cps) and faster diffusion rate of solvent enhance the encapsulation efficiency. As the ratio of polymer increased particle size, encapsulation efficiency was also increased. This is because of the saturation concentration of organic phase increased with viscosity at maximum ratio which helps to enlarge the size and a maximum encapsulation with a homogeneous matrix. Viscosity also influenced percentage yield and encapsulation efficiency of recovered microparticles. As polymer concentration increased, the binding capacity or matrix forming competency of polymer with drug also increased. Due to this the maximum amounts of drug get entrapped in polymeric core and give more encapsulation and percentage yield of recovered microparticles in higher drug polymer ratio than lower ratio [17]. Ethylcellulose also acts as a self-emulsifier to stabilize this emulsion. The emulsion droplets containing Propranolol hydrochloride get transformed into solid state due to Ethylcellulose hydrophobic property and acetone evaporation during stirring. This evaporation rate was maintained by stirring at 1000 rpm and 25°C temperature.  Table 1 shows the influence of drug-polymer ratio, surfactant, and its concentration on particle size, percentage yield, and encapsulation efficiency of different formulation trials.

**Figure 1:**
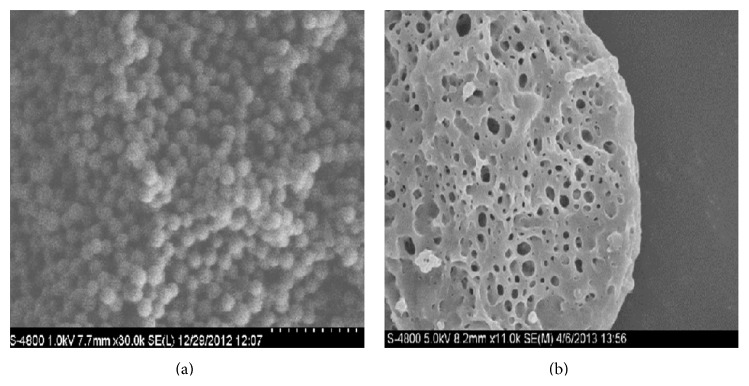
Surface morphology of recovered microparticles.

The obtained results concluded that the fact that the particle sizes were directly proportional to polymers concentration may be due to increase in viscosity of the internal phase, and inversely proportional the surfactant concentration may be due to the formation of new surfaces for the small emulsion globules. Formulation prepared using Ethylcellulose 300 cps showed higher encapsulation efficiency (96.7 ± 0.5) than those prepared using Eudragit RS 100 (83.7 ± 0.6) and RL 100 (89.7 ± 0.6). It was also found that release of drug is slower than formulation prepared from Eudragit polymers. Formulations F4, F6, and F12 which are prepared using higher drug  to polymer ratio with 0.4% surfactant concentration showed drug release of 59.08 ± 0.2, 75.10 ± 0.4, and 92.9 ± 0.3 percent, respectively, at 12 h. All recovered microparticles were spherical in shape and slightly porous in nature (Figure 1). This porous nature of microparticles is responsible for sustained release of drugs, because the polymer concentration may help to enhance the holding capacity for drug and surfactant decreased the interfacial tension between aqueous drug solution and polymeric organic solution.

Drug polymer interaction was determined by comparing the IR spectra of Propranolol hydrochloride loaded Ethylcellulose microparticles with the IR spectrum of pure drug.

**Figure 2:**
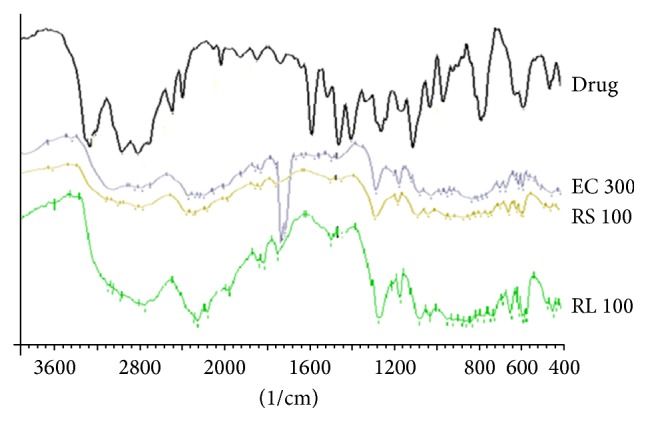
FTIR Spectra.

As shown in Figure 2, Propranolol hydrochloride gives peaks in the IR spectrum nearby at 2977 cm^−1^ due to the presence of a secondary amine group, 3372 cm^−1^ due to the hydroxyl group (secondary), the aryl alkyl ether shows a stretching band at 1354.64 cm^−1^ and the peak at 782 cm^−1^ due to a-substituted naphthalene. Frequencies of functional groups of pure drug remained intact or overlapped by polymer in a physical mixture containing polymer. Hence, there was no major interaction between the drug and polymer used in the study.

**Figure 3:**
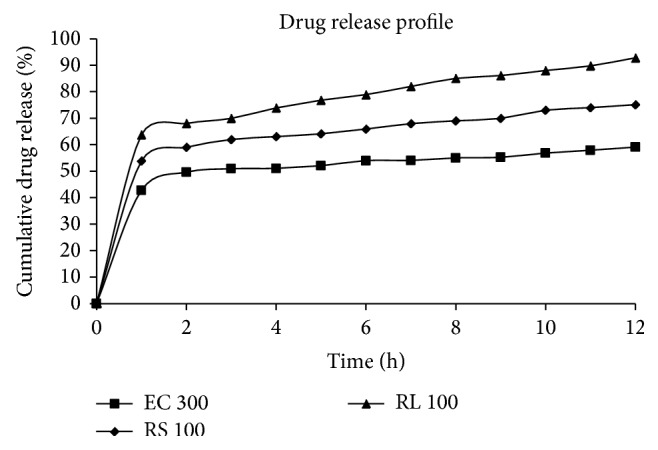
Release profile of Propranolol hydrochloride from some selected formulations.

Figure 3 illustrates* in vitro* release of F4, F6, and F12 formulations, by representing the cumulative percentage of drug released.

The formulation F4 was more sustained than formulations F8 and F12, because the maximum number of particles may extend the time to release the drug from F4 formulation. In F4 formulation prepared by using Ethylcellulose polymer and 0.4%, v/v span 80 surfactant was used which gives larger size particles than F8 and F12 formulation. At the end of 12 hours F4 formulation released 59.08 ± 0.2, F8 released 75.10 ± 0.4, and F12 released 92.9 ± 0.3 percent propranolol hydrochloride. Drug bursts at the first hour of the formulations F4, F8, and F12 are 42.8 ± 0.4, 53.9 ± 0.4, and 63.8 ± 0.4, respectively; it may be due to the drug present at the surface of particles. The burst release effect may be due to the adsorption of the drug on the surface of the particles or due to concentrating the drug at the surface of the particles because insufficient concentration or ineffectual surfactant was unable to encapsulate drugs at the core of particles and drug moved towards the interface of both phases. The release kinetics of all selected formulations is explained in Table 2. The obtained results revealed that the most sustained F8 formulation best fitted in Higuchi kinetics.

Formulations F4, F8, and F12 all show Higuchi model. It describes the release of drugs from an insoluble matrix as a square root of time dependent process based on Fickian diffusion [18].

In Higuchi or square root kinetics, drug diffuses at a comparatively slower rate as the distance for diffusion increases. From all above evaluations, it was concluded that the prepared microparticles were successfully sustained for 12 h. Thus from all these results it was discovered that Ethylcellulose 300 cps viscosity range polymer can be used to formulate sustained release microparticles at different ratios.

## 4. Conclusion

From the above results, it was concluded that Propranolol hydrochloride was successfully encapsulated into Ethylcellulose and Eudragit microparticles using O/O hydrophobic emulsion solvent evaporation method. Span 80 was more suitable surfactant with a concentration of 0.4%, v/v. The 1 : 7 drug-polymer ratio obtained the highest encapsulation and sustained the propranolol hydrochloride for 12 h. It follows Higuchi model release kinetics; therefore this dosage form maintains the drug level in therapeutic window which may help to minimize the side effects and minimize the frequency of dose which improve patient compliance.

## Figures and Tables

**Table 1:** Batch yield, entrapment efficiency, drug content, and size of prepared microparticles.

Polymer grade	Code	Ratio	% yield	Encapsulation efficiency	Drug content	Particle size (*μ*m)
EC 300	F1	1 : 1	91.6 ± 0.5	83.7 ± 0.6	45.9 ± 0.3	139 ± 13
	F2	1 : 3	94.0 ± 0.4	87.7 ± 0.6	23.7 ± 0.5	261 ± 24
	F3	1 : 5	95.8 ± 0.3	93.7 ± 0.6	16.7 ± 0.5	388 ± 32
	F4	1 : 7	98.6 ± 0.6	96.7 ± 0.5	12.8 ± 0.4	580 ± 56

RS 100	F5	1 : 1	57.6 ± 0.6	68.6 ± 0.6	60.0 ± 0.3	133 ± 14
	F6	1 : 3	63.9 ± 0.2	73.7 ± 0.5	29.0 ± 0.3	246 ± 38
	F7	1 : 5	71.6 ± 0.7	77.8 ± 0.7	18.6 ± 0.5	368 ± 43
	F8	1 : 7	76.8 ± 0.3	83.7 ± 0.6	16.8 ± 0.5	464 ± 44

RL 100	F9	1 : 1	62.6 ± 0.5	73.9 ± 0.8	59.3 ± 0.2	142 ± 28
	F10	1 : 3	67.6 ± 0.6	79.7 ± 0.6	29.9 ± 0.3	263 ± 42
	F11	1 : 5	85.2 ± 0.2	84.7 ± 0.6	16.9 ± 0.3	379 ± 49
	F12	1 : 7	89.0 ± 0.1	89.7 ± 0.6	12.9 ± 0.4	508 ± 58

**Table 2:** *In vitro *release kinetics study.

Formulation code	*r* ^2^ (regression coefficient)			
	Zero order	First order	Higuchi	Hixon-Crowell
F4	0.8744	0.9046	**0.9280 **	0.8953
F8	0.9657	0.9828	**0.9856 **	0.9793
F12	0.9870	0.9783	**0.9933 **	0.9929
